# Identifying functional movement deficits in mild to moderate adolescent idiopathic scoliosis: a cross-sectional study

**DOI:** 10.3389/fped.2026.1681171

**Published:** 2026-04-10

**Authors:** ShuangFei Liu, Jiehang Xiao, Biao Chen, Haitao Fan, Tianqi Yao

**Affiliations:** 1 School of Public Teaching, Ningbo Polytechnic, Ningbo, China; 2 Faculty of Kinesiology and Physical Education, University of Toronto, Toronto, ON, Canada; 3 Ningbo Institute of Sports Science, Ningbo, China; 4 Department of Orthopaedics, The First Affiliated Hospital of Ningbo Universitry, Ningbo, China; 5 National Institute of Sports Medicine, Beijing, China

**Keywords:** adolescent, Cobb angle, functional movement screen, idiopathic scoliosis, movement deficit

## Abstract

**Objective:**

Currently, research on adolescent idiopathic scoliosis (AIS) focuses more on corrective treatment and prevention, and few studies have been conducted on movement deficits within this population. Study utilized a functional movement screening (FMS) method to test and evaluate movement characteristics of patients with AIS.

**Methods:**

Functional movement performance was evaluated in adolescents with mild-to-moderate AIS using the FMS protocol. Kendall's Tau correlation analysis was employed to examine the relationship between FMS scores and Cobb angle magnitude. Based on the number of curves and directions of Cobb angles, three main classifications of scoliosis were found within participants: thoracolumbar double-curve, thoracic single-curve, and lumbar single-curve.

**Results:**

The mean composite FMS score for all the participants (*n* = 32) was 11.78 ± 1.5, while double-curve group scored slightly lower (11.8 ± 1.0) than single-curve group, although the difference was not significant (*P* > 0.05). Among individual FMS movements, Trunk Stability Push-Up (TSP) demonstrated the lowest score (0.88 ± 0.79). In sex-based comparison, males performed significantly better in left Hurdle Step (HS) and TSP (*P* < 0.05), whereas females scored significantly higher in right Shoulder Mobility (SM) and bilateral Active Straight Leg Raise (ASLR) (*P* < 0.05). Importantly, Kendall's Tau correlation analysis revealed no significant correlation between Cobb angle magnitude and total FMS scores (r = 0.136, *P* > 0.05) in the overall cohort. The only exception was Rotary Stability (RS), demonstrating a weak but significant positive correlation with Cobb angle (r = 0.336, *P* < 0.05).

**Conclusions:**

Adolescents with mild to moderate AIS exhibit composite FMS scores consistently below the clinical reference threshold of 14, indicating generalized functional movement deficits. Importantly, the generalized deficits exist independently of radiographic severity of the AIS, indicating the necessity for functional assessments in addition to radiographic analysis. Meanwhile, these limitations were particularly noticeable in trunk stability, suggesting a need for targeted motor control training.

## 1 Introduction

Adolescent idiopathic scoliosis (AIS), a complex three-dimensional (which is defined as coronal curvature, altered sagittal curvature, and transverse section) rotational (3-Dimensional, 3D) structural deformity of the spine (1) which is common in children from 10 years of age to skeletal maturity (2). The pathomechanism of scoliosis is unknown in approximately 90% of the scoliosis population, which explains why the condition is also known as idiopathic scoliosis (3). Based on the Scoliosis Research Society (SRS) criteria, the Adam's forward bend test (FBT) and the Bunnell scoliometer are used to measure the scoliosis of patients. Angle of trunk rotation (ATR) was measured by using a scoliometer for census, and usually the ATR threshold is between 4° and 7°. The most commonly used value of 5° was empirically determined to be the ATR threshold (4). In the analysis of the images irradiated in the posterior-anterior position by x-rays (36-inch film, from the head to the pelvis, including the hip and femoral head), patients who develop a Cobb angle greater than 10°or more with vertebral rotation can be identified as having AIS (5). They can be categorized as single-curve and double-curve based on the number of curves (5, 6).

In addition, Cobb angle has been graded as mild (10° ≤ Cobb angle < 25°), moderate (25° ≤ Cobb angle < 45°), and severe (45° ≤ Cobb angle) from the selection of clinical treatment modalities (1, 7). Usually there are two types of treatment for AIS: surgical and non-surgical; and in the non-surgical cases the mild and moderate ones are treated with Schroth therapy (8), brace therapy (9), Chinese osteopathy (10), Pilates (11), Suspension therapy (12), and core strength (13); extending moderate-intensity physical activity while reducing sedentary time (14). In the surgical cases, surgical procedures such as anterior vertebral tethering (AVT) are generally required (15). The majority of patients with mild and moderate AIS are corrected by non-surgical methods, which is also a hot topic of current research.

Functional Movement Screen (FMS) is assessed by a 7-movement total score (16). It works as a basis for assessing movement scores and injury risk thresholds for athletes and non-athletes, but it has been controversial. Currently, research suggests that FMS critical scores should be applied across different sports like basketball, softball, running, martial arts, and swimming, etc. (17–22), and different occupations like firefighters, rangers, police, infantry (23–26). While the composite FMS score ≤ 14 has been associated with injury risk in athletic populations (17), the threshold has not been validated in AIS populations. Despite that there are differences among the FMS critical scores, the simplicity, non-invasiveness, wide range of applications, stable reliability, efficiency, and accuracy of its 7 action testing procedure have been well received and recognized by scholars (27), which indicates the FMS assessment can be applicable to the AIS population.

Currently, the global prevalence of AIS ranges between 0.47% and 5.2%, with children aged 13–18 years accounting for 1%–3% of the total as well as a male-to-female ratio of 2:1, and the gender gap increases with the enlargements in disease severity and age (28, 29). Some studies have proved that in AIS populations, the neuro-osseous development of the spine and spinal cord is not synchronized (30), and there could be shoulder asymmetry, scapular prominence, unequal waist circumferences, and lower limb length differences (4); abnormal gait and posture (31); also accompanied by body asymmetry, muscle imbalance, loss of flexibility and back pain (11). In terms of proprioception, a study has proved that the physical appearance of the AIS population produces certain deformities, such as asymmetry of the paraspinal and trunk muscles, contributing to one of the factors that destabilize the sense of balance of the body and proprioceptively decrease the patients' abilities to detect the kinematic changes of body portions, especially in the lower extremities and the back (32). Moreover, a negative correlation was found between the muscle tested next to the thoracic spine bending site by electromyography (EMG), and the cross-sectional area of the erector spinae and left quadratus lumborum (5). Patients with AIS have also been found to be associated with spinal rotation and altered sagittal physiologic curvature, and even rotation and tilt of the ribs and pelvis and abnormalities of the surrounding soft tissues (3). However, there is currently little research on whether these changes in body shape affect motor abilities in the AIS populations, with only few studies reporting that moderate and vigorous exercise can reduce the likelihood of having scoliosis get worse by 30% in children with AIS (33). Hence, the assessment of functional motor abilities in patients with AIS is currently understudied in the literature. Based on that, three hypotheses were formulated in this study: (1) functional movement patterns would vary across different curve types (single-curve vs. double-curve); (2) FMS scores would be associated with the severity of the Cobb angle; and (3) the majority of AIS patients would exhibit composite FMS scores below the standard clinical threshold of 14. The primary objective of this study was to characterize the functional movement profiles of adolescents with mild-to-moderate AIS and to determine whether functional deficits differ according to curve pattern (single-curve vs. double-curve) or radiographic severity (Cobb angle).

## 2 Materials and methods

### 2.1 Study procedures

**Figure 1:**
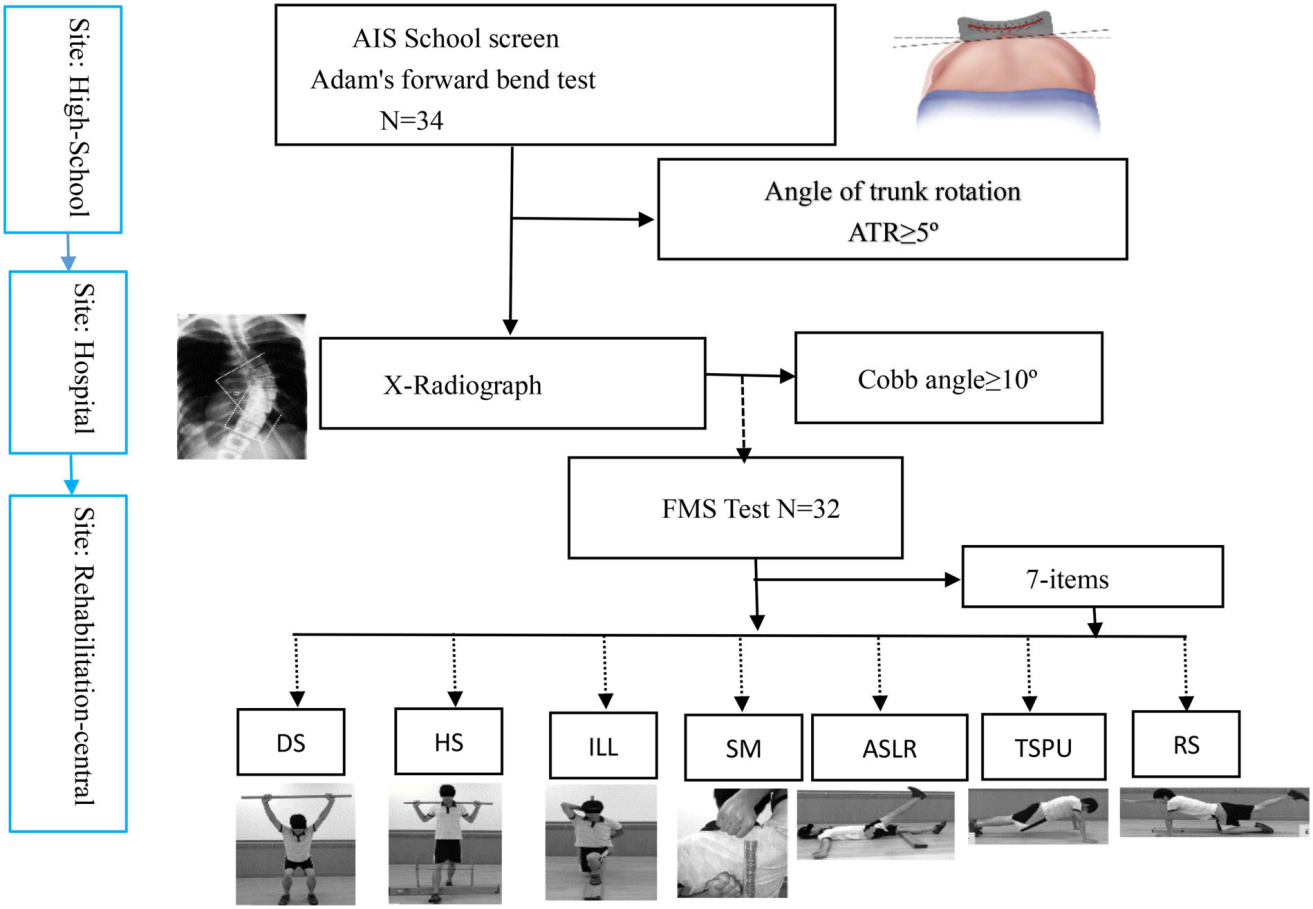
Flowchart of screening, confirmation of diagnosis and FMS testing of AIS patients. DS, deep squat; HS, Hurdle step; ILL, inline lunge; SM, should mobility; ASLR, active straight leg raise; TSP, trunk stability push-up; RS, rotary stability.

This study is based on a campus-based activity from February 2024 to February 2025 on a scoliosis screening public welfare program in Ningbo, Zhejiang Province from China. Before the test, parents and students were signed with informed consent. The project was carried out mainly from the census of the campus scoliosis ATR program to the confirmation of the clinical Cobb angle. Participants who were diagnosed with AIS will have the test in the orthopedic rehabilitation department of the rehabilitation clinic. The whole experimental process shown in Figure 1 was supported by all levels of the government and people from all walks of life. The study complied with the Declaration of Helsinki and was approved by the Ethics Commission of the First Affiliated Hospital of Ningbo University, and the acceptance number is 2023-R075-02.

### 2.2 Subjects of the study

In this study, 34 high school students of 24 females and 10 males, aged 15–16 years, who were previously diagnosed with AIS were selected to participate in the test. The inclusion criteria were: (1) diagnosis of AIS with Cobb angle between 10° and 45°; (2) age between 10 and 18 years. The exclusion criteria included: (1) history of spinal surgery or other musculoskeletal disorders affecting movement; (2) congenital spinal disorders; (3) family history of spinal and other medical conditions; (4) significant pain on the test day. Patients currently undergoing brace treatment were included, but they were required to remove the brace during testing. However, participants who had engaged in systematic functional rehabilitation (e.g., intensive Schroth therapy) within the past 3 months were excluded in order to minimize confounding factors. In addition, there was a significant difference in height and weight of the males compared to the females, and the mean value of the ATR angle was greater than 5 degrees (4). However, during the testing process, one male and one female student quitted due to academic plans, and hence the data of 32 patients was eventually collected for this study with 23 females and 9 males.

### 2.3 Classification of cobb angle on radiographs of AIS patients

**Figure 2:**
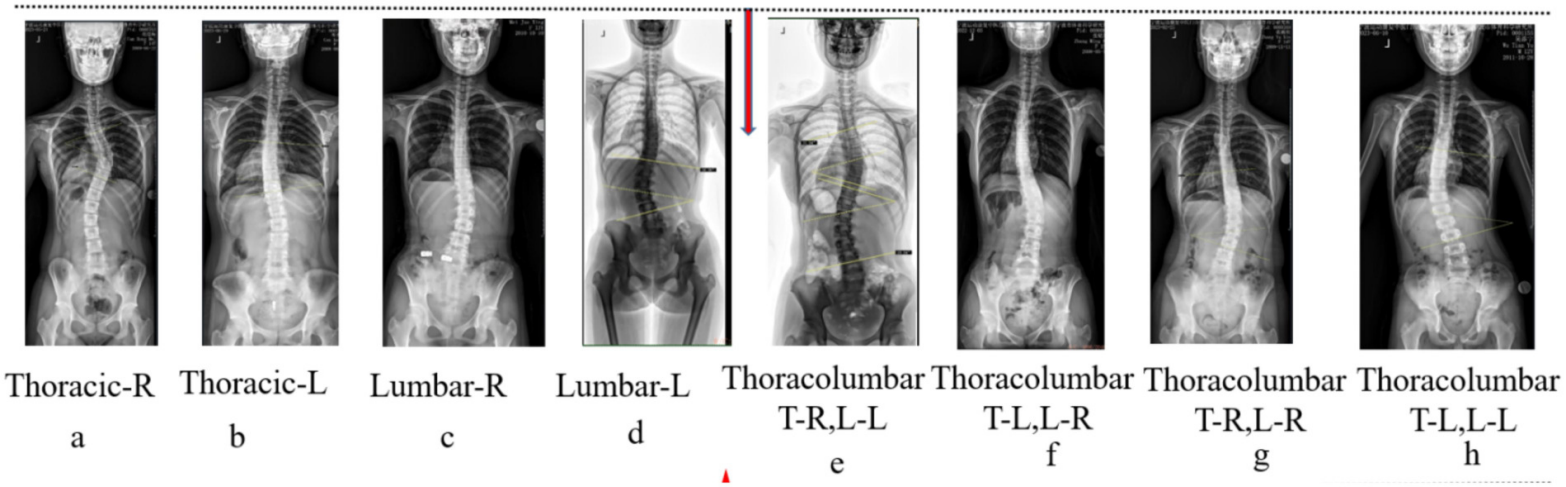
Single-curve bending **(a,b,c,d)** and double-curve bending **(e,f,g,h)**. R, right; L, left; T-R, thoracic-right; T-L, thoracic-left; L-R, lumbar-right; L-L, lumbar-left.

The number of curvatures and direction the Cobb angles of scoliosis were classified by 3 clinical spine doctors on the radiographs, and if there were any abnormalities, the 3 doctors negotiated the classification. According to the classification of Cobb angle referring to the current clinical treatment correction method, mild scoliosis is 10° ≤ Cobb angle < 25°; moderate 25° ≤ Cobb angle < 45° (1); and also the scoliosis types can be classified into triple-curve (e.g., cervical, thoracic, and lumbar) bending, double-curve (e.g., thoracolumbar) bending, and single-curve bending (4). Moreover, based on the orientation of scoliosis on the x-ray film, they can also be divided into the following 8 categories (Figure 2), which are the most prevalent types among the AIS patients. Nevertheless, in this study, only 5 of them (a, c, d, e g) were found from the participants due to the limited sample size and therefore the cases of b, f, and h will not be discussed.

### 2.4 FMS program test determination basis and process

The Movement Function Screening consists of 7 movements including Deep Squat (DS), Hurdle Step (HS left and right), Inline Lunge (ILL left and right), Active Straight Leg Raise (ASLR left and right), Shoulder Mobility (SM left and right), Trunk Stability Push-Up (TSP) and Rotary Stability (RS). Each movement scale was judged on a 4-point scale from 0 to 3. A score of 0 was given if any pain occurred during the test, 1 indicated that the movement could not be performed (34), 2 indicated that the subject had to perform the movement through motor compensation, and 3 referred to that the movement was accurately represented. In addition, the lowest score from the left and right sides in the same movement was written in the total score (16). For example, if the left straight lunge was scored as 3, while the right straight lunge was scored as 2, the lowest score should be taken as the right side score which is 2 (22). Finally, all these scores were summed up into a composite score ranging from 0 to 21. The test was conducted by three physiotherapists on the weekends, and there was no warm-up during this. Crucially, three raters were blinded to the participants' radiographic details like Cobb angle magnitudes and curve types during the testing session, preventing scoring bias. Each participant was tested individually in order to eliminate the influence of study and practice on FMS measurements, and each movement was repeated three times during the test. In addition, the participants were asked to report the feelings of any pain or discomfort while performing the movement. All testing sessions were video recorded to facilitate the reviewing and ensure scoring accuracy. The assessment was simultaneously evaluated by three physiotherapists, and to minimize subjective bias and enhance scoring consistency, the final score for each movement was determined by taking the median score of the three raters, excluding the highest and lowest ratings.

### 2.5 Statistical analysis

The baseline of participants was analyzed using descriptive statistics mean plus or minus standard deviation (Mean ± SD); functional movement screening scores were non-normally distributed using a non-parametric Mann–Whitney Test; in addition, Kendall's Tau correlation was used to evaluate the relationship between FMS scores and Cobb angle severity among different curve patterns (single-curve and double-curve), and the correlation coefficients were interpreted as weak correlation (r < 0.19), low correlation (0.20–0.39), moderate correlation (0.40–0.59), strong correlation (0.60–0.79), and very strong correlation (r > 0.8) (35). The above statistical analyses were performed using IBM SPSS for Windows Version 25.0 (IBM Corp., Armonk, NY, USA) software, and the symbol * represents significance at *P* < 0.05.

## 3 Results

### 3.1 Characterization of subjects' demographic information and distribution of cobb angles

The total number of participants is 32, with the females of 23 and the males of 9. There was a significant difference between males and females in terms of weight and BMI (*P* < 0.05). Specifically for BMI, the mean level of females (18.1 ± 1.3) was below 18.5 which is considered as low-BMI, while the mean value of males (19.3 ± 3.2) was higher than 18.5, belonging to the normal-BMI range. In ATR screening, the males' ATR values (9.5 ± 1.9) were slightly lower than the females' (9.6 ± 2.5) (Table 1).

**Table 1:** characteristics of the study population.

Characteristics	Females (*n* = 23)	Males (*n* = 9)	ALL(*n* = 32)
Age (±; SD)	14.8 ± 1.0	14.7 ± 0.9	14.8 ± 1.0
Body weight (±; SD) (kg)	48.7 ± 4.7	58.1 ± 12.0^a^	51.4 ± 8.5
Height (±; SD) (cm)	164.1 ± 4.9	173.1 ± 5.3	166.6 ± 6.4
BMI	18.1 ± 1.3	19.3 ± 3.2^a^	18.4 ± 2.0
ATR (°)	9.6 ± 2.5	9.5 ± 1.9	9.6 ± 2.3

n, sample size; ±; SD, mean value and standard deviation; BMI, body mass index. FMS, function movement screen; ATR, angle of trunk rotation.

^a^ significant difference between males and females.

From the classification of subjects' x-ray Cobb angles, there were 15 cases of single-curve bending including both thoracic and lumbar bending and were accounted for 46.9% of the total. Among them, 60% were thoracic-right bending, 13.3% were lumbar-right bending, and 26.7% were lumbar-left bending. Besides, there were also 17 cases of double-curve bending with 52.9% of which are the right thoracolumbar ipsilateral (same-side) bending, and 47.1% of them are the heterolateral (different-side) bending of thoracic-right-lumbar-left (Table 2). Due to the limited sample size in specific subgroups (e.g., *n* = 2 in lumbar-right), only descriptive statistics of Cobb angles (Mea*n* ± SD) are presented in Table 2.

**Table 2:** Scoliosis location distribution by cobb angle.

Curve Pattern	Location	Convexity	*n*	Cobb angle (°) (Mean ± SD)
Single-curve	Thoracic	Right	9	17.6 ± 5.7
	Thoracic	Left	N/A	N/A
	Lumbar	Right	2	10.5 ± 0.7
	Lumbar	Left	4	14.8 ± 1.5
Double-curve	Thoracolumbar (ipsilateral)	Right (T & L)	9	T: 18.22 ± 4.9, L: 20.7 ± 3.8
		Left (T & L)	N/A	N/A
	Thoracolumbar (heterolateral)	Right (T) & Left (L)	8	T: 24.6 ± 9.5, L: 23.8 ± 9.4
		Left (T) & Right (L)	N/A	N/A

n, number; T, thoracic; L, lumbar; N/A, not available.

### 3.2 Sex differences in FMS scores

In the analysis of FMS sub-scores, it was shown that males scored significantly higher than females on left HS and TSP (*P* < 0.05); however, the females' right SM and bilateral ASLR scores were significantly higher than males (*P* < 0.05). It was also found that the left and right RS scores were low in both males and females. Even though females scored slightly higher in total FMS scores (11.9 ± 1.1) than males (11.4 ± 2.2), the difference was not significant (*P* > 0.05) (Table 3).

**Table 3:** Scoring of individual test items of the functional movement screen (FMS).

FMS movements	Male (*n* = 9)	Female (*n* = 23)	Combined (*n* = 32)
Deep Squat	1.44 ± 0.72	1.7 ± 0.55	1.6 ± 0.61
Hurdle Step	1.78 ± 0.44	1.87 ± 0.34	1.84 ± 0.36
Right	1.78 ± 0.44	1.99 ± 0.11	1.94 ± 0.24
left	2.2 ± 0.44^a^	1.87 ± 0.34	1.97 ± 0.4
Inline lunge	1.67 ± 0.7	1.61 ± 0.65	1.63 ± 0.66
Right	1.78 ± 0.44	1.74 ± 0.619	1.75 ± 0.56
left	1.67 ± 0.5	1.78 ± 0.6	1.75 ± 0.56
Shoulder Mobility	2.3 ± 0.71	2.52 ± 0.59	2.47 ± 0.62
Right	2.56 ± 0.72	2.74 ± 0.45^a^	2.69 ± 0.53
left	2.44 ± 0.73	2.57 ± 0.59	2.53 ± 0.62
Active straight leg raise	2.11 ± 0.61	2.48 ± 0.59^a^	2.38 ± 0.61
Right	2.22 ± 0.66	2.6 ± 0.58^a^	2.5 ± 0.62
left	2.11 ± 0.6	2.7 ± 0.55^a^	2.5 ± 0.62
Trunk stability push-up	1.22 ± 0.97^a^	0.74 ± 0.68	0.88 ± 0.79
Rotary stability	0.89 ± 0.33	1.0 ± 0.30	0.97 ± 0.3
Right	0.89 ± 0.33	1.0 ± 0.36	1.0 ± 0.35
left	0.89 ± 0.33	1.0 ± 0.36	1.0 ± 0.35
Total FMS score	11.4 ± 2.2	11.9 ± 1.1	11.78 ± 1.5

^a^ significant difference between male and female.

### 3.3 Comparison of different scoliosis sites, cobb angles and BMI with FMS

**Figure 3:**
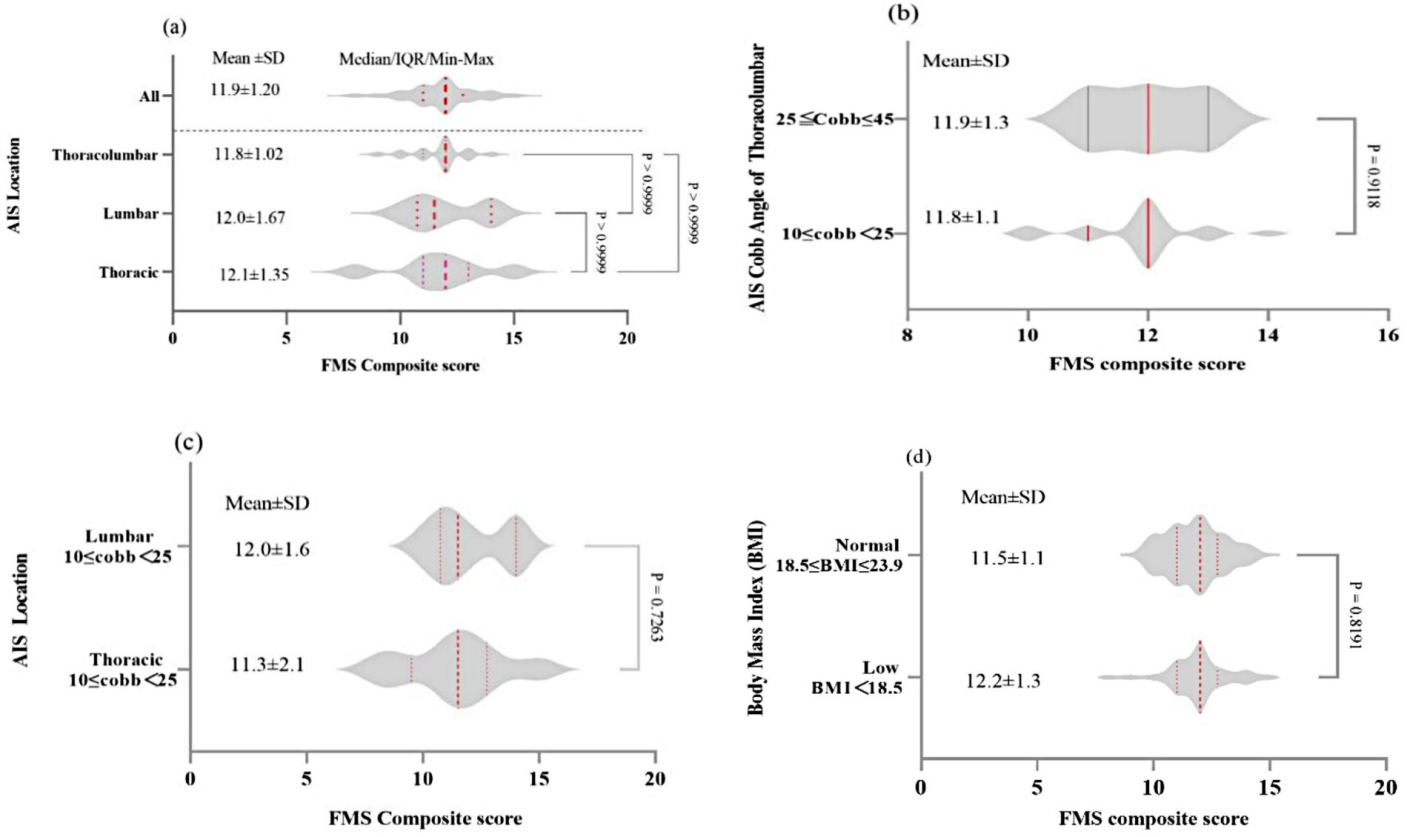
Relationship between scoliosis site, cobb angle and BMI in AIS: **(a)** Violin plots of FMS composite scores by AIS location (thoracic, lumbar, thoracolumbar, and overall groups); **(b)** Comparison of FMS scores by thoracolumbar Cobb angle; **(c)** Comparison of FMS scores between lumbar and thoracic groups; **(d)** Comparison of FMS scores by BMI category (normal vs. low BMI).

Comparisons of total FMS scores exhibited no significant differences between single-curve and double-curve groups (*P* > 0.05), while the mean score of the double-curve group (11.8 ± 1.0) was slightly lower than those of single-curves, with the mean values of 12.0 ± 1.67 in lumbar and 12.1 ± 1.35 in thoracic region (Figure 3a). In terms of AIS severity (Cobb angle), no significant differences were found between patients with mild (10° ≤ Cobb angle < 25°, 11.8 ± 1.1) and moderate (25° ≤ Cobb angle < 45°, 11.9 ± 1.3) scoliosis within double-curve group (*P* > 0.05, Figure 3b). While both single-curve groups (thoracic and lumbar) fell within the category of mild (10° ≤ Cobb angle < 25°), there was no significant difference between two groups (*P* > 0.05, Figure 3c), and the mild lumbar single-curve group had a slightly higher FMS score (12.0 ± 1.6) compared to the mild thoracic group (11.3 ± 2.1). Finally, in consistency with the Cobb angle findings, BMI classifications of normal and low had no significant impact on FMS composite scores (*P* > 0.05, Figure 3d), while the patients with low BMI scored higher (12.2 ± 1.3) than normal BMI (11.5 ± 1.1).

### 3.4 Correlation between FMS scores and maximal cobb angle by curve pattern

Due to the limited sample sizes in directional subgroups (e.g., *n* = 2 in lumbar-right and *n* = 4 in lumbar left), correlation analysis was restricted to the boarder classification based on curve numbers (single-curve vs. double-curve) to ensure statistical reliability. As shown in Table 4, Kendall's Tau analysis revealed that in All group (*n* = 32), no significant relationship between Cobb angle and the FMS composite score (r = 0.136, *P* > 0.05), and similarly, in the sub-groups of Single-curve (*n* = 15) and Double-curve (*n* = 17), none of them demonstrated significant relationships between Cobb angle and the FMS composite scores (r = 0.245, *P* > 0.05; r = −0.070, *P* > 0.05, respectively). In individual FMS movements, most of them (DS, HS, ILL, SM, ASLR, TSP) did not show significant relationships with Cobb angle no matter in All, Single-curve, or Double-curve groups (*P* > 0.05), with the only exception of RS showing a significant positive relationship with Cobb angle magnitude (r = 0.336, *P* < 0.05), even though the relationship was weak (Table 4).

**Table 4:** Kendall's Tau correlation coefficients between FMS scores and maximal cobb angle by curve pattern.

FMS movement	All (*n* = 32)	Single-curve (*n* = 15)	Double-curve (*n* = 17)
Deep Squat	−0.003	0.012	−0.084
Hurdle Step	0.221	0.340	0.144
Inline Lunge	−0.089	−0.074	−0.091
Shoulder Mobility	0.274	0.090	0.302
Active Straight Leg Raise	0.019	−0.113	−0.103
Trunk Stability Push-Up	−0.032	0.378	−0.231
Rotary Stability	0.336^a^	0.200	0.307
Total FMS score	0.136	0.245	−0.070

^a^ significant correlation. Correlations were calculated by selecting the maximal Cobb angle in double-curve group.

## 4 Discussion

### 4.1 Main findings summarization

The primary objective of this study was to investigate and characterize FMS patterns in adolescents with mild to moderate AIS. Importantly, the study found that the entire participants demonstrated an average FMS composite score of 11.78 ± 1.5. Among the individual tasks, TSP and RS yielded the lowest scores (0.88 ± 0.79 and 0.97 ± 0.3 respectively), indicating a prevalent deficit in core stability and motor control of trunk muscles. Besides, the analysis revealed no significant correlation between Cobb angle and the FMS composite score in all 32 participants (r = 0.136, *P* > 0.05), and the similar results were found in single-curve group (r = 0.245, *P* > 0.05) and double-curve group (r = −0.070, *P* > 0.05). Despite that in all participants, a weak significant positive correlation was found between Cobb angle and the individual FMS scores in RS (r = 0.336, *P* < 0.05), the rest of individual FMS movements still appeared non-significant correlations with Cobb angle in all the groups, reflecting that the movement deficits of AIS populations could be independent of Cobb angle. In terms of sex differences, the composite FMS scores were similar between males (11.4 ± 2.2) and females (11.9 ± 1.1), but in individual movements the significant differences were observed: males performed significantly better in left HS (2.2 ± 0.44) and TSP (1.22 ± 0.97) compared to females (1.87 ± 0.34 and 0.74 ± 0.68 respectively, *P* < 0.05), whereas females had significantly higher scores in right SM (2.74 ± 0.45) and bilateral ASLR (2.48 ± 0.59) than males (2.56 ± 0.72 and 2.11 ± 0.61 respectively, *P* < 0.05), indicating that males generally performed better in stability tasks while females were generally good at tasks that require mobility and flexibility.

### 4.2 Movement deficits and the clinical threshold

In the present study, a mean composite FMS score of 11.78 ± 1.5 was notably lower than the critical score of 14 out of 21 suggested by previous injury prediction models (36, 37), and even though the cross-sectional design does not include direct injury prediction, the existing literature suggests that scores below this threshold are associated with suboptimal movement patterns and potential vulnerability to musculoskeletal injury (19, 37, 38). The findings also confirm that adolescents with AIS face significant limitations in performing basic functional movements especially in trunk stability (TSP and RS) based on individual FMS scoring data. This aligns with the understanding that AIS is not merely a static skeletal deformity but a complex condition involving three-dimensional trunk asymmetry (39), which also destabilizes the body's center of gravity and compromises proprioception (32). The generalized low scores observed in the participants suggest that even mild-to-moderate spinal curvature is sufficient to disrupt the kinetic chain, necessitating comprehensive functional screening beyond routine radiographic monitoring.

### 4.3 Independence of movement deficit from cobb angle

A key finding of this study is the non-significant correlation revealed between Cobb angle magnitude and the FMS composite scores. This contradicts the intuitive assumption that a higher curvature severity could linearly correspond to greater movement deficits, aligning with previous findings that radiographic severity does not necessarily predict patient-reported outcomes (33). A meaningful implication can also be yielded from the finding: the movement deficits in AIS patients may happen independently of radiographic severity, which means an adolescent with a mild (10° ≤ Cobb angle < 25°) scoliosis might possibly exhibit the same degree of movement deficits as the one with a moderate (25° ≤ Cobb angle < 45°) curvature. The independence of generalized movement deficits in AIS population further supports the view that neuromuscular control deficits in AIS may be a primary or parallel feature rather than solely a secondary consequence of the spinal curvature (30).

While almost all the FMS sub-scores exhibited non-significant relationships with Cobb angle across all the groups, RS was shown to be the only exception that demonstrated a weak but significant positive correlation with Cobb angle magnitude (r = 0.336, *P* < 0.05). The plausible explanation is that, as scoliosis progresses, the increased trunk asymmetry may lead to body imbalance (13), while spine might develop increased structural rigidity to maintain whole-body stability and postural alignment (30, 40), and this mechanism could thereby reduce excessive movement during the RS task, leading to a slightly higher RS score compared to lower Cobb angle cases that might show greater hypermobility. Therefore, the compensatory stiffness may conceal underlying movement deficits by limiting range of motion, creating a passive stability. Collectively, the general non-significant correlation across most tasks and the unique compensatory significant relationship observed in RS share a common clinical implication that radiographic severity alone is a poor indicator of degree of an AIS patient's movement deficit. Instead, an independent functional assessment is needed to further determine the movement quality.

### 4.4 Trunk stability as a primary contributor to movement deficits

In the study, TSP and RS demonstrated the lowest performances (0.88 ± 0.79 and 0.97 ± 0.3 respectively) among the seven FMS movements. This finding points to a specific weakness in core stability. It is believed that trunk asymmetry in AIS can lead to paraspinal muscle imbalances, specifically between the multifidus and erector spinae (5). Such imbalances can impair the neuromuscular coordination required to maintain a neutral spine during dynamic movements (41). Moreover, from an anatomical perspective, the rectus abdominis primarily synergistically works with back muscles like erector spinae to support the TSP task, resisting spinal extension, while internal and external obliques essentially provide rotational torque required for RS activity. The poor TSP and RS scores reinforce the recommendation that rehabilitation for AIS should prioritize core stabilization exercises (40), and strengthening core stabilizers (including the transvs. abdominis, internal and external obliques, rectus abdominis, multifidus, and diaphragm) could be essential not only for curve management but also for improving the patient's capability to perform fundamental moves safely (8).

### 4.5 Impact of sex and curve pattern

Consistent with general adolescent population trends, sex differences were observed in individual FMS tasks. Males performed better in stability and power tasks (TSP, HS), while females scored higher in flexibility tasks (SM, ASLR). However, the composite scores did not differ significantly between sexes, suggesting that movement deficits are shared equally among male and female AIS patients. Furthermore, the analysis suggested no significant difference in FMS scores between single-curve and double-curve groups, suggesting that the presence of any significant scoliosis itself is enough to disrupt body's movement qualities and lead to lower FMS scores, regardless of the specific curve patterns.

### 4.6 Clinical implications

The non-significant relationship between Cobb angle severity and FMS scores suggests that radiographic severity alone is insufficient to determine the movement deficits of an adolescent with AIS. An adolescent with a stable, mild Cobb angle may still possess significant motor control deficits that could predispose them to pain or reduced physical activity participation (42–44). These findings highlight the potential benefit of integrating functional movement assessments, such as the FMS, into the routine clinical evaluation of AIS. Such a comprehensive approach will allow clinicians to identify specific motor deficits (particularly in trunk stability) and design rehabilitation programs (e.g., targeted motor control training) to address these functional limitations, thereby promoting safer participation in physical activities (45).

## 5 Conclusion

This study evaluated 32 AIS patients with Cobb angles ranging from 10° to 38° to characterize their functional movement profiles. The analysis demonstrates that generalized functional movement deficits are prevalent among adolescents with mild-to-moderate AIS, as evidenced by an average composite FMS score of 11.78 ± 1.5, consistently falling below the clinical threshold of 14. These functional limitations appear to be systemic and independent of curve patterns, given that no significant differences in composite FMS scores were observed between single-curve and double-curve subgroups. Furthermore, the analysis reveals a dissociation between anatomical structure and functional performance, as FMS scores showed no significant linear correlation with Cobb angle magnitude, suggesting that radiographic severity alone does not dictate motor control competence. Notably, the RS task demonstrated a weak but significant positive correlation with curve severity, suggesting that compensatory stiffness may conceal underlying instability in curves with higher Cobb angles. Collectively, these findings emphasize that AIS patients' radiographic results alone are insufficient to determine their movement deficits, underscoring the complexity and independence of such deficits in AIS populations.

## 6 Limitations

This study was not without limitations. First, the sample size (*n* = 32) was relatively small, even though the number was sufficient for descriptive analysis for the entire cohort and broader classifications (single-curve and double-curve). As a result, it restricted the statistical power for subgroup comparisons (e.g., lumbar-right), and it was unable to perform correlation analyses for these detailed classifications. Second, this cross-sectional study did not incorporate a healthy control group. Even though the established FMS clinical threshold of 14 was used to evaluate the movement deficits in AIS population, the absence of a non-AIS control group limits the precise quantification of functional deviations relative to healthy peers. Third, although in clinical implications it was suggested to integrate functional movement assessments into the routine clinical AIS evaluations to identify specific functional deficits, there was no prospective injury tracking. Therefore, no empirical analyses derived from realistic injury data tracking can be used to predict injury probability in AIS populations. Finally, there were methodological limitations related to assessment protocols: participants were not allowed to warm up, which deviated from the original FMS protocol, and although FMS scoring was conducted by licensed physiotherapists, formal inter-rater reliability statistics (e.g., Cohen's Kappa or ICC) were not calculated in this study. Furthermore, generalized joint laxity (e.g., Beighton score) and skeletal maturity (e.g., Risser's classification) were not assessed. Hypermobility is a common feature in AIS (46) and could theoretically inflate scores in mobility-biased tasks like SM, and therefore the lack of a laxity adjustment may conceal certain functional stability deficits. Meanwhile, as the risk of curve progression and spinal flexibility varies significantly with skeletal maturity, analyzing FMS scores across different Risser grades could provide deeper insights into how growth phases impact functional motor control. Future research should incorporate these meaningful biological measurements as well as longitudinal study designs with healthy controls to help further investigate the injury predictability of FMS in AIS.

## Data Availability

The original contributions presented in the study are included in the article/Supplementary Material, further inquiries can be directed to the corresponding author/s.
